# CircRNA hsa_circ_0008500 Acts as a miR-1301-3p Sponge to Promote Osteoblast Mineralization by Upregulating PADI4

**DOI:** 10.3389/fcell.2020.602731

**Published:** 2020-12-11

**Authors:** Qiaoli Zhai, Yi Zhao, Linping Wang, Yan Dai, Peiqing Zhao, Xinxin Xiang, Kui Liu, Wenyan Du, Wenxiu Tian, Baoye Yang, Tao Li, Lianqing Wang

**Affiliations:** ^1^Center of Translational Medicine, Zibo Central Hospital, Shandong University, Zibo, China; ^2^School of Stomatology, Shandong University, Jinan, China

**Keywords:** hsa_circ_0008500, RUNX2, PADI4, miR-1301-3p, osteoblast mineralization

## Abstract

Circular RNAs (circRNAs) are regarded as pivotal regulators in bone metabolism. However, the role of circRNAs in osteoblast mineralization remains largely unknown. Herein, we explored the expression profiles of circRNAs in 4 groups of osteoblasts with varying mineralization processes. Hsa_circ_0008500 (circ8500), which is upregulated in the RNA-seq data, is sifted through 194 candidate circRNAs in osteoblasts during mineralization. We characterize the features of novel circRNAs and find that the elevated expression of circ8500 promotes osteoblast mineralization. Mechanistically, circ8500 contains a critical binding site for miR-1301-3p. We further show that circ8500 competitively binds miR-1301-3p to abolish its suppressive effect on peptidyl arginine deiminase 4 (PADI4). PADI4 works as a binding partner of RUNX2 and stabilizes its protein expression levels by inhibiting the ubiquitin-proteasome pathway. This work provides new insights on the circRNA patterns in osteoblasts and the role of PADI4 in matrix mineralization.

## Introduction

Osteoblast differentiation, which is an essential process of bone formation, is orchestrated at different stages by coding and non-coding factors (Brunner et al., 2011). Osteoblast matrix mineralization is a key stage in bone differentiation, and inhibition of the process results in severe bone diseases such as osteogenesis imperfecta and osteoporosis (Wang et al., 2018; Ibrahim et al., 2019). Osteoporosis represents a huge economic burden on the United States, with annual costs estimated to be $17 billion (Estrada et al., 2012). Matrix mineralization is a rigorously administrated process, invoking osteogenic genes and signaling pathways (Wang et al., 2019). Alkaline phosphatase (ALP) activity, a hallmark of the osteoblast phenotype, is considered critical to the initiation of mineralization (Henthorn et al., 1992). Collagen type I is a major matrix component that provides the required substrate for mineral deposition (Marcus et al., 2013). Among the non-collagenous matrix proteins, RUNX2 plays vital roles in osteoblast mineralization (Vimalraj et al., 2015). RUNX2 acts as trans activator of bone cells. Osteocalcin (OCN), the target gene of RUNX2, is a marker of differentiated osteoblasts, and its expression is positively correlated to matrix mineralization (Bruderer et al., 2014).

Circular RNAs (circRNAs), microRNAs (miRNAs), and other non-coding RNAs plays crucial roles in cell activities (Guttman and Rinn, 2012; Chatterjee and Sengupta, 2019). CircRNAs are characterized by covalently closed continuous loop structures without 5’ to 3’ polarity and a polyadenylated tail (Memczak et al., 2013). A growing number of circRNAs have been discovered in various eukaryotes with the integration of next-generation sequencing and bioinformatics (Chioccarelli et al., 2019; Ragusa et al., 2019). With accumulating knowledge of features and functions of circRNAs, these are now regarded as pivotal regulators in various biological processes. Interestingly, circRNAs modulation downstream oral amino acid supplementation improves sperm vitality and motility, showing the relationship between differentially expressed circRNAs and drug therapy (Manfrevola et al., 2020). To date, only a small number of circRNAs have been functionally characterized. At the molecular level, three important roles of circRNAs have been identified: (i) modulate miRNA activity (Hansen et al., 2013; Lasda and Parker, 2014), (ii) regulate alternative splicing (Ashwal-Fluss et al., 2014) and (iii) interact with RNA-binding proteins (Du et al., 2016). The first function of circRNAs, described as competitive endogenous RNAs (ceRNAs), has been extensively studied in various diseases. For example, circHIPK3, a well-known circRNA, acts as a miRNA sponges and is involved in bladder cancer development (Li et al., 2017). circHRCR protects the heart from pathological hypertrophy by targeting miR-223 (Wang et al., 2016).

In osteogenesis, circRNAs have been shown to be significantly regulated, and several bone-related circRNAs were identified (Zhang et al., 2019). A recent study has indicated that circRNA hsa_circ_0074834 could promote osteogenesis in bone mesenchymal stem cells by acting as a ceRNA for miR-942-5p (Ouyang et al., 2019). Another study, revealed that circCDr1as, acting as a sponge of miR-7, promotes osteogenic differentiation of periodontal ligament stem cells (PDLSCs) (Li et al., 2018). In MC3T3-E1, which are preosteoblast derived from murine cells, circ19142 and circ5846 play collaborative roles during osteoblast differentiation (Qian et al., 2017). Although sporadic reports have shown that circRNAs are crucial to osteogenic differentiation, their roles in osteoblast mineralization are largely unknown. Zheng and colleagues briefly investigated the expression profile of circRNAs during osteogenic differentiation of PDLSCs using array analysis (Zheng et al., 2017). However, the roles and mechanisms of circRNAs in mineralization have not been extensively clarified.

In this study, we investigated the expression profiles of circRNAs and mRNAs during matrix mineralization of hFOB 1.19 cells using high throughput RNA sequencing (RNA-seq). Then, a significantly upregulated circRNA, has_circ_0008500 (circ8500), is initially identified. We found that ectopic expression of circ8500 distinctly promoted osteoblast matrix mineralization via sponging miR-1301-3p to upregulate *PADI4* expression. The functional importance of PADI4 stabilizing RUNX2 was further illustrated in osteoblast matrix mineralization. This study provides new insights into circRNA patterns in osteoblasts and the role of PADI4 in matrix mineralization.

## Materials and Methods

### Cell Culture and Treatments

Human embryonic kidney (HEK) 293T and human osteoblast hFOB1.19 cells were obtained from Stem Cell Bank of the Chinese Academy of Sciences (Shanghai, China). The HEK293T cells were cultured in DMEM supplemented with 10% FBS at 37°C with 5% CO_2_. The hFOB1.19 cells were cultured in DMEM/F12 supplemented with 10% FBS at 33.5°C with 5% CO_2_. To detect changes in cell behavior during mineralization, hFOB 1.19 cells were plated at 1 × 10^6^ cells/well into 6-well plates for alkaline phosphatase assays or Von Kossa staining, 2 × 10^5^ cells/well into 24-well plates for osteocalcin assays, 5 × 10^6^ cells into 10-cm dishes for Western blot analysis. The cells were cultured (DMEM/F12 supplemented with 10% FBS) at 39.5°C for mineralization. In the *PADI4* inhibition experiments, cells were treated with 100 μM Cl-amidine (Sigma-Aldrich, St. Louis, MO, United States) for 24 h. In the mineralization experiment, 50 μM Cl-amidine was used to inhibit PADI4. DMSO (Sigma-Aldrich), the solvent of the Cl-amidine, was used as the control.

### RNA Isolation, Library Synthesis, and RNA Sequencing

At different time points of induction of hFOB 1.19 cells [i.e., before (day 0) and after (days 3, 6 and 9)], osteoblasts were collected and used in RNA-Seq analysis. Total RNA was extracted using TRIzol reagent (Tiangen, Beijing, China) according to the manufacturer’s protocols. The purity and concentration of total RNA were assessed using a NanoDrop 2,000 spectrophotometer (Thermo Fisher Scientific, Waltham, MA, United States), and RNA integrity was checked by a Bioanalyzer 2100 system (Agilent Technologies, Santa Clara, CA, United States).

For library construction, 5 μg of RNA from each sample was prepared. Epicenter Ribo-Zero^TM^ rRNA Removal Kit (Epicenter Biotechnologies, Madison, WI, United States) was used to remove rRNA, and ethanol precipitation was performed to clean up rRNA-free residue. For mRNA library construction, linear RNAs were treated by RNase R (Epicenter Biotechnologies), which was not used in the library construction of circRNAs. The sequencing libraries were generated using NEBNext Ultra II Directional RNA Library Prep Kit (New England Biolabs, Ipswich, MA, United States) according to the manufacturer’s protocol. Transcriptome sequencing on an Illumina HiSeq X Ten platform was conducted at Novogene Bioinformatics Technology Co., Ltd. (Beijing, China).

### Bioinformatics Analysis

Raw data in fastq format were first treated with a custom perl script. Reads containing adapter, ploy-N, and low-quality reads from raw data were removed. The Q20, Q30, and GC contents of the selected reads were calculated (Supplementary Table S1).

Reference genome and gene model annotation files were downloaded from genome website^1^. The reference genome index was built using Bowtie v.2.0.6,55 and the paired-end clean reads were aligned to the reference genome with TopHat v.2.0.9. The circRNAs were detected and identified using find_circ and CIRI2. Differential expression analysis for the control and infection groups was performed using DESeq2 (v. 1.6.3). *p*-values were adjusted using the Benjamini-Hochberg method.

### PCR and qPCR Analyses

RNA from separate nuclear and cytoplasmic fractions was isolated using PARIS^TM^ Kit (Thermo Fisher Scientific). Total RNA was extracted and subjected to reverse transcription using the PrimeScript 1st Strand cDNA Synthesis Kit (TaKaRa, Otsu, Japan). For mRNA and circRNA, a mixture of random and oligo d(T) primers were employed in reverse transcription. For miR-1301-3p, random primers and 5′-GTCGTATCCAGTGCAGGGTCCGAGGTATTCGCACTGG ATACGACGAAGTC-3′were used in the reaction system. gDNA was isolated using a TIANamp Genomic DNA Kit (Tiangen).

The Premix Taq DNA Polymerase (TaKaRa) was used for PCR. The cDNA and gDNA PCR products were analyzed using 2.5% agarose gel electrophoresis. Quantitative real-time PCR was performed using the SYBR Premix Ex Taq II (TaKaRa). Relative mRNA and circRNA levels were normalized to *GAPDH*. In the control group, the gene expression was calibrated as 1. For miRNAs, U6 was used as the internal control. The primers used in PCR and qPCR analyses are summarized in Supplementary Table S2. All the primers span multiple introns and/or exons, and were validated by melting curve analysis.

### Alkaline Phosphatase (ALP) Activity Assay

ALP activity in hFOB1.19 cells and culture media were measured. hFOB 1.19 cells were plated to 6-well plates (1 × 10^6^ cells/well) and cultured at 33.5°C for 2 days (day 0). The hFOB1.19 cells were grown at 33.5°C for 2 days (day 0), and the medium was replaced with fresh DMEM/F12 followed by an additional 12 days of culture at 39.5°C. The medium and cells were collected every 2 days from days 0 to 12. ALP activity in the medium and lysates were quantified by an ALP Assay Kit (Beyotime). Cells were washed twice with PBS, and lysed with lysis buffer (Beyotime, Shanghai, China). Five microliter lysate were removed for determination of protein concentration. Fifty microliter lysate were incubated with ALP substrate (50 μL), and the solution was incubated for 10 min. Absorption was measured at 405 nm using a Multiskan FC (Thermo Fisher Scientific), and conversion to enzyme activity was made using a*p*-nitrophenol standard absorption curve.

### Osteocalcin Detection Assay

Circ8500 overexpression and knockdown constructs were transfected into hFOB 1.19 cells using lentivirus. hFOB 1.19 cells transfected with blank vectors were used as the controls. The cells were grown at 39.5°C for differentiation. The medium was collected and replaced with fresh DMEM/F12 every 2 days. Osteocalcin ELISA kits (Abcam, Cambridge, United Kingdom) were used to detect OCN levels. Centrifuge cell culture media at 2,000 × g for 10 min to remove debris. Collect supernatants and assay. Add 50 μL of all sample or standard to appropriate 96-wells. Add 50 μL of the Antibody Cocktail to each well. Seal the plate and incubate for 2 h at room temperature on a plate shaker set to 400 rpm. After washing each well with Buffer PT, add 100 μL of TMB Development Solution to each well and incubate for 10 min in the dark on a plate shaker set to 400 rpm. Add 100 μL of Stop Solution to each well. Record the OD at 450 nm by Multiskan FC.

### Von Kossa Staining

Von Kossa staining was performed to determine osteoblast mineralization. hFOB 1.19 cells were seeded at a density of 1 × 10^6^ cells per 6-well plate. At time points 4, 8, and 12 days, the cells were fixed in 4% paraformaldehyde for 10 min. The cells were then washed thrice with distilled water and stained with 2% AgNO_3_ (Aladdin, Shanghai, China) for 60 min under UV light. The cells were washed three times with distilled water and incubated with 5% Na_2_S_2_O_3_ (Aladdin) for blocking for 5 min. After dehydration with alcohol, the cells were cleared and mounted in DPX Mountant (Merck Millipore, Darmstadt, Germany).

### Vector Construction and Cell Transfection

To stably express circ8500, the AluSq2 and AluSz circular elements in pcDNA3.1(+) CircRNA Mini Vector (Liang and Wilusz, 2014) were cloned into lentiviral vector pLVX-puro, and the recombinant vector (pLVX-CircRNA) was used as negative control (circ-NC). The full-length cDNA of circ8500 was amplified from the *DLG1* transcript (NM_001098424) in hFOB1.19 cells and then cloned into pLVX-CircRNA. To knock down circ8500, siRNA targeting the back-splice junction site of circ8500 and a scrambled sequence were synthesized by Invitrogen (Thermo Fisher Scientific). Then, the sequence of shRNA against circ8500 and the negative control were inserted into the pLKO.1 vector, which were then named as sh-circ and sh-NC, respectively. The miRNA mimics and inhibitors were synthesized by GenePharma (Shanghai, China).

The sequences of circ8500 and PADI4 3′-UTR and their relevant mutants without miR-1301-3p binding sites were PCR amplified. All of the sequences were subcloned into a luciferase reporter vector pGL3-Promoter (Promega, Madison, WI, United States) at the *Xba*I site using an In-Fusion^®^ HD cloning kit (TaKaRa). The constructs were termed circ8500-WT, circ85001-Mut, PADI4 3′-UTR-WT, and PADI4 3′-UTR-Mut, respectively.

*PADI4* transcripts (GenBank Acc. No. NM_012387) with or without 3′-UTR were PCR amplified and cloned into pCDNA3.1 (+) (Thermo Fisher Scientific) or pLVX-puro Vector. pcDNA3.1-HA-PADI4 and pCMV-HA-Ubiquitin were provided by Ye Zhang (Peking Union Medical College). Human *RUNX2* (GenBank Acc. No. NM_001024630) coding region was obtained from hFOB 1.19 cDNA and it cloned into a pCMV-3Tag6 vector (Agilent Technologies). The human osteocalcin-luc plasmid was constructed according to an earlier report (Piscopo et al., 2009). All of the constructs were confirmed by direct sequencing. The primers used for construction are listed in Supplementary Table S3.

Cell transfections were performed with Lipofectamine 3000 (Thermo Fisher Scientific) according to the manufacturer’s protocols. For stable transfection, virus particles were produced by transfecting HEK293T cells with lentivirus vectors and packaging vectors (psPAX2, pMD2G), and then hFOB1.19 cells were infected with virus-containing supernatants.

### Luciferase Assays

HEK-293T or hFOB1.19 cells were seeded in 24-well plates for 12 h before transfection. The cells were co-transfected with a mixture of 500 ng luciferase reporter plasmids, 25 ng pRL-TK (*Renilla* luciferase) vector, and miRNA mimics in the indicated experiments. Forty-eight hours after transfection, luciferase activity was measured using a dual luciferase assay kit (Promega) in accordance with the manufacturer’s protocols. The relative activities of luciferase were normalized to the *Renilla* luciferase values, and then fold changes were calculated.

### RNA Immunoprecipitation (RIP)

RIP experiments were conducted with a Magna RIP RNA-binding protein immunoprecipitation kit (Merck Millipore). hFOB1.19 cells were harvested 48 h after transfection of miR-1301-3p mimics or miR-NC and lysed in RNA lysis buffer. The cell lysates were incubated with magnetic beads conjugated with antibodies [anti-Argonaute2 (AGO2) or IgG antibody, Cell Signaling Technology, ıDanvers, MA, United States] at 4°C for 4 h. The beads were treated according to the manufacturer’s protocols. The co-precipitated RNA was purified and detected by qRT-PCR.

### RNA Pull-Down

The 3′-end biotinylated circ8500 probe (5′-TGAGAAGAAGCTTCAGGTCG-3′) and control probe (5′-GG GTTTCAATATTGGCTTCA-3′) were synthesized by Invitrogen. hFOB1.19 cells, stable transfected with circ8500 or negative control, were lysed and incubated with specific probes. The biotin-coupled RNA complex in cell lysates was pulled down by streptavidin-coated magnetic beads (Thermo Fisher Scientific). The abundance of miR-1301-3p in the bound fraction was evaluated by qRT-PCR.

### Co-immunoprecipitation and Western Blotting

For western blot analysis, cells were lysed in RIPA (Beyotime) with a protease inhibitor mixture (Roche, Basel, Switzerland). Proteins were separated on SDS-PAGE gels under reducing conditions and transferred onto a PVDF membrane (0.22 μm, Merck Millipore). After blocking for non-specific binding, the membranes were probed with antibodies specific to HA (1:5,000, sc-40, Santa Cruz Biotechnology, Dallas, TX, United States), FLAG (1:5,000, MBL, Beijing, China), PADI4 (1:1,000, Merck Millipore), or RUNX2 (1:1,000, Sigma-Aldrich) overnight at 4°C, followed by incubation with corresponding secondary antibodies (1:10,000, MBL) for 1 h at room temperature. Bands were detected by a Tanon 5200 Multi System (Tanon, Shanghai, China) or developed on X-ray films (XBT, Kodak, Rochester, NY, United States).

hFOB 1.19 cells, induced for 3 days, and transfected HEK293T cells were lysed for 0.5 h in IP lysis buffer (Thermo Fisher Scientific) with a protease inhibitor mixture (Roche). The cell lysates were centrifuged at 13,000 *g* for 5 min at 4°C. The supernatant was incubated with anti-FLAG (M185-3L, MBL) or anti-RUNX2 (D1H7, Cell Signaling Technology) antibody for 4 h and then with protein-G agarose beads (Roche) overnight at 4°C with gentle shaking. The beads were collected and washed five times with IP lysis buffer. The immunoprecipitated proteins were analyzed by western blotting with FLAG, HA, RUNX2 and PADI4 antibodies, separately (Wang et al., 2018; Zhai et al., 2020).

In all of the assays, protein GAPDH (primary antibody, 1:5,000, sc-2578, Santa Cruz Biotechnology) was used as the internal control.

### *In vivo* Protein Decay Assay

The 293T cells were transfected transiently with the indicated expression plasmid(s) or treated with Cl-amidine (100 μM) for 24 h. Before harvesting, the cells were cultured with cycloheximide (20 μg/mL, Sigma-Aldrich) for 0, 30, 60, or 120 min. Cells were harvested at different time points and total lysates were immunoprecipitated with the indicated antibodies. The immunoprecipitated proteins were analyzed by western blotting.

### Statistical Analysis

The results are presented as mean ± SD. Statistical analysis was performed by GraphPad Prism 5.0, and differences between two experimental groups were assessed by the Student’s *t*-test. Differences with a *p* < 0.05 were considered to be statistically significant.

## Results

### Characteristics of Circ8500 During Osteoblast Mineralization

**FIGURE 1:**
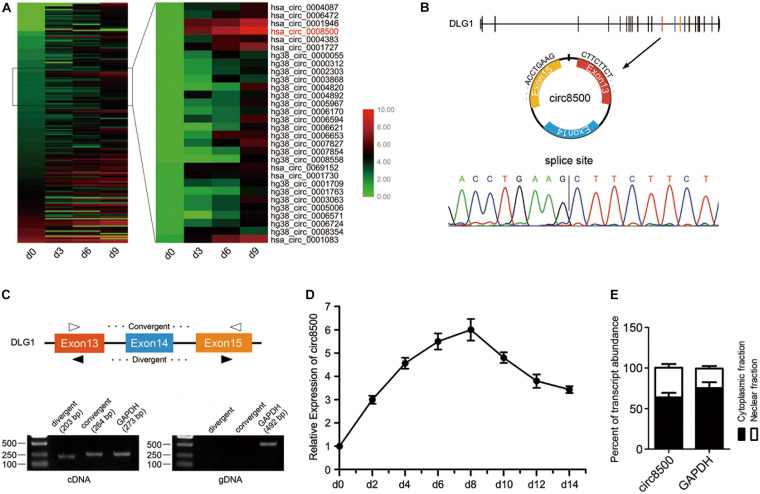
CircRNA expression profile reveals circ8500 expression during osteoblast mineralization. **(A)** Heat map showing the circRNAs that are differentially expressed during osteoblast mineralization. A total of 194 circRNAs were analyzed, with sequence read counts ≥ 20 at any of the four time points. Circ8500 is shown in red. The data for the analysis was from GSE148203_circRNA in GEO. **(B)** The existence of circ8500 was validated in hFOB 1.19 cells by RT-PCR. Using divergent or convergent primers, circ8500, was amplified using cDNA as template but not using genomic DNA (gDNA). GAPDH was used as internal control. **(C)** Schematic illustration showing circularization of DGL1 exons 13–15, forming circ8500 (black arrow). Chromatogram showing the ‘head-to-tail’ splicing site of circ8500. **(D)** Relative expression of circ8500 in hFOB 1.19 at different mineralization time points. **(E)** Levels of circ8500 in the nuclear and cytoplasmic fractions of hFOB1.19 cells. Error bars **(D,E)** represent s.d. (*n* = 3).

To understand the expression profiles of circRNAs and mRNAs during osteoblast mineralization, we conducted RNA-seq in four paired hFOB 1.19 cells. During osteoblast mineralization, a total of 8,464 regulated circRNAs were identified, of which 6,332 circRNAs were upregulated and 2,132 circRNAs were downregulated (Supplementary Figures S1A–E). KEGG pathway analysis revealed that pathways in osteoblast mineralization are pathways related to cancer, which include cell proliferation, apoptosis and differentiation (Supplementary Figure S1F). In addition, 1,633 differentially expressed mRNAs were also identified, with 1,027 mRNAs upregulated and 606 mRNAs downregulated (Supplementary Figure S2). To explore crucial circRNAs that are involved in matrix mineralization, we selected circRNAs whose sequence read counts were ≥ 20 at any of the four groups. A total of 194 circRNAs were sifted through the RNA-seq data for further analysis. We then selected several persistent differential circRNAs, of which has_circ_0001946 and has_circ_0008500 were significant different candidates (Figure 1A). Has_circ_0001946, also known as CDR1as or ciRS-7, has been extensively characterized in previous studies (Barrett et al., 2017; Chen et al., 2020; Hanniford et al., 2020). We investigated the functional importance of has_circ_0008500 (circ8500) in the subsequent experiments.

Circ8500 was presumed to be derived from exons 13 to 15 of the *DLG1* gene, which is located at chr3:196831773–196846401, and finally formed a circular transcript of 381 nt according to the annotation of circBase^2^. To verify the existence of circ8500, the cDNA of circ8500 was amplified in hFOB1.19 cells and confirmed by direct sequencing (Figure 1B). However, the spliced junction in circ8500 could be produced by *trans*-splicing or genomic rearrangements. Thus, to rule out these possibilities, we designed convergent and divergent primers to amplify circ8500. Using cDNA and gDNA (genomic DNA) from hFOB 1.19 cell lines as templates, circ8500 was only amplified in cDNA, and no amplification product was observed in gDNA (Figure 1C). Using real-time PCR, we analyzed the expression profiles of circ8500 during osteoblast mineralization. It was revealed that the expression of circ8500 increased at the beginning of osteoblast differentiation and peaked on day 8. It subsequently decreased, although it remained at a higher level (Figure 1D). To explore the role of circ8500 in osteoblasts, we analyzed the cellular localization of circ8500 by qPCR. The results showed that the circ8500 is transcript preferentially located in the cytoplasm (Figure 1E). These results confirm the characteristics of circ8500 as a circRNA and suggest that it facilitates matrix mineralization.

### Circ8500 Promotes Osteoblast Mineralization

**FIGURE 2:**
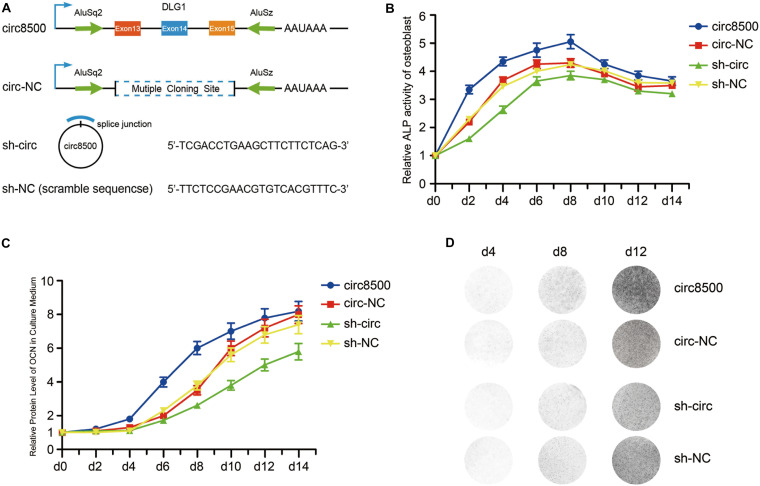
Circ8500 promotes osteoblast mineralization. **(A)** Schematic illustration of circ8500 expression and shRNA vectors. hFOB 1.19 cells were separately stably transfected with circ8500, circ-NC, sh-NC, and sh-circ. The cells were routinely cultured at 33.5°C and then transferred to 39.5°C to maintain their mineralized state. **(B)** The effect of circ8500 on alkaline phosphatase activity in hFOB 1.19 cells at different mineralization time points. **(C)** The effect of circ8500 on OCN during osteoblast mineralization was examined by ELISA. **(D)** Von Kossa staining showing the mineralization of hFOB1.19 extracellular matrix with different circ8500 expression levels. Error bars **(B,C)** represent s.d. (*n* = 3).

To explore the biological function of circ8500 in mineralization progression, the overexpression vector of circ8500 and the RNAi (sh-circ) vector against circ8500 was cloned into the lentiviral vector (Figure 2A). The results showed that circ8500 was overexpressed or knocked down in hFOB 1.19 cells after stable transfection (Supplementary Figure S3A). Furthermore, we found that sh-circ could knockdown circ8500, but it had no effect on DLG1 mRNA expression in hFOB 1.19 cells (Supplementary Figure S3B).

Growth curves performed by CCK8 assays revealed that ectopic expression of circ8500 significantly suppresses osteoblast proliferation, whereas downregulation of circ8500 promotes cell growth (Supplementary Figure S3C). These also suggested that circ8500 inhibits osteoblast growth and may benefit the mineralization process. To confirm our presumption, we first detected the effect of circ8500 on ALP activity during matrix mineralization. In the first 8 days of osteoblast mineralization, ALP activities were significantly enhanced by the upregulation of circ8500 and markedly impaired by the downregulation of circ8500. In the later stages of osteoblast mineralization, circ8500 had little effect on ALP activity (Figure 2B). Then, we examined the expression of OCN, a mineralization marker. To assess changes in OCN resulting from circ8500, we performed ELISA using the hFOB cell supernatant. ELISA revealed that overexpression of circ8500 markedly increased the secretion of OCN from days 6 to 10 of mineralization, whereas knocking down of circ8500 imparted a delayed effect (Figure 2C). The effect of circ8500 on calcium deposition was further visualized by Von Kossa staining, which indicated mineralization on day 8. Circ8500 overexpression promoted calcium deposition and decreased with knockdown of circ8500 (Figure 2D). These results suggest that circ8500 enhances the mineralization of osteoblasts.

### Circ8500 Functions as a Sponge for miR-1301-3p

**FIGURE 3:**
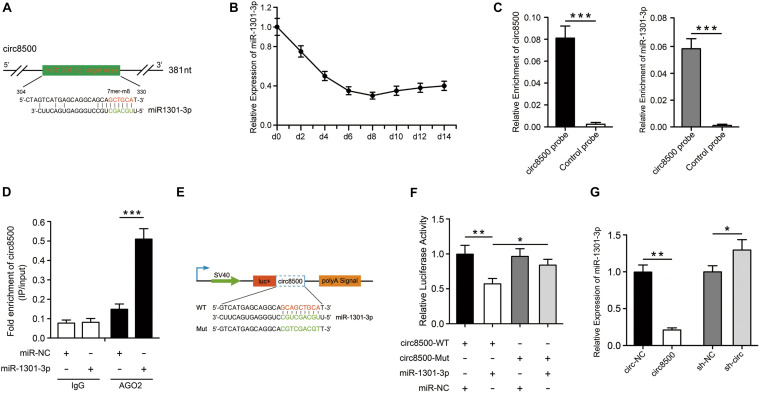
Circ8500 acts as sponge for miR-1301-3p. **(A)** The miR-1301-3p binding site on circ8500 as predicted by RegRNA 2.0. **(B)** Relative expression of miR-1301-3p in hFOB 1.19 cells during osteoblast mineralization. **(C)** Enrichment of miR-1301-3p by circ8500 examined using an RNA pull-down assay. **(D)** The interaction of circ8500 and miR-1301-3p detected by RIP. **(E)** Schematic illustration of circ8500 wild-type and mutant luciferase reporter vectors. **(F)** Relative luciferase activities in HEK-293T cells co-transfected with circ8500-WT or Mut and miR-1301-3p mimics or miR-NC. **(G)** Relative expression of miR-1301-3p as detected by qPCR after transfection with the indicated vectors. The data are presented as the mean value ± SD (*p < 0.05, **p < 0.01, ***p < 0.001).

To investigate the molecular mechanism underlying circ8500, we predicted the potential targets of circ8500 using RegRNA2.0^3^. The bioinformatic analysis showed that circ8500 possesses a 7mer-8m target site for miR-1301-3p (Figure 3A). We examined the expression of miR-1301-3p during matrix mineralization and found that it decreased to a valley value approximate 1/3 of what it was before (Figure 3B), which suggests that miR-1301-3p playes a regulatory role in mineralization. Given that most of the circ8500 are located in the cytoplasm, it may function as the sponge of miR-1301-3p. In addition, we investigated whether circ8500 could directly bind to miR-1301-3p.

To verify the binding of circ8500 and miR-1301-3p, we performed an RNA pull-down assay with specific biotin-labeled circ8500 probes. A specific enrichment of circ8500 and miR-1301-3p was detected by qRT-PCR in the circ8500 probe group compared with the control (Figure 3C). miRNA is known to play a regulatory role by combing Argonaute 2 (AGO2). Hence, we used an AGO2 immunoprecipitation assay to determine whether circ8500 serves as a platform for AGO2 and miR-1301-3p. Figure 3D shows that circ8500 in hFOB 1.19 cells was abundantly pulled down by miR-1301-3p. To elucidate the binding capability of miR-1301-3p to circ8500, we constructed luciferase reporters containing wild-type and mutated putative binding sites of circ8500 (Figure 3E). Luciferase assays showed that the luciferase activity of the circ8500 wild-type reporter significantly decreased when transfected with miR-1301-3p mimics compared with the control or mutant reporter (Figure 3F). Moreover, we found that ectopic expression of circ8500 leads to a marked decrease in miR-1301-3p expression, and silencing of circ8500 could significantly increase miRNA expression in hFOB1.19 cells (Figure 3G).

Our results reveal that circ8500 functions as a miR-1301-3p sponge and we investigated the functional target gene of miR-1301-3p in the subsequent study.

### PADI4 Is Targeted by miR-1301-3p and Indirectly Regulated by Circ8500

**FIGURE 4:**
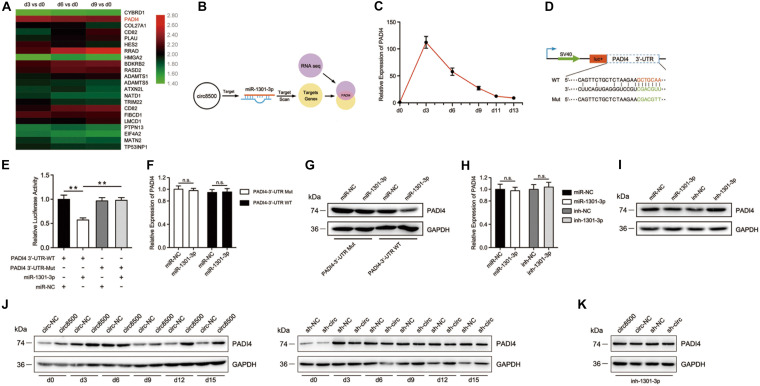
PADI4 is targeted by miR-195-5p and is indirectly regulated by circ8500. **(A)** Heat map showing the upregulation of PADI4 in mRNA expression profiles during osteoblast mineralization. The data for the analysis came from GSE148203_mRNA in GEO. **(B)** The schematic illustration showing that PADI4 is the potential target of miR-1301-3p. **(C)** Relative expression of *PADI4* was detected by qPCR in hFOB 1.19 cells at different mineralization time points. **(D)** Schematic illustration of PADI4 3′-UTR of the wild-type and mutant luciferase reporter vectors. **(E)** Relative luciferase activities in HEK-293T cells co-transfected with PADI4 3′-WT or Mut and miR-1301-3p mimics or miR-NC, respectively. **(F,G)** Relative expression of PADI4 was detected by qPCR and western blotting in HEK-293T cells co-transfected with the indicted vectors and miR-1301-3p mimics or miR-NC. **(H)** and **(I)** Relative mRNA and protein levels of PADI4 were detected in hFOB 1.19 cells after transfection with miR-1301-3p, miR-NC, inh-1301-3p, or ihn-NC. **(J)** Relative protein levels of PADI4 at different time points of mineralization were detected in hFOB 1.19 cells after stable transfection with circ8500, circ-NC, sh-NC, or sh-circ. **(K)** miR-1301-3p mimic was transfected to different hFOB 1.19 cells with circ8500, circ-NC, sh-NC, or sh-circ, and PADI4 was detected by western blotting. Data are represented as mean value ± SD (n.s., not significant, **p < 0.01).

First, we selected the top 50 potential target genes of miR-1301-3p that were predicted by TargetScanHuman^4^ (Supplementary Table S4). Then, we searched across the mRNA sequencing data and identified genes that were shared between the two data sets. *PADI4* is the only gene hitting the target (Figures 4A,B). PADI4, a kind of deiminase, converts both arginine and monomethyl-arginine to citrulline in the presence of Ca^2+^ (Nakashima et al., 1999). Citrullination modification of substrates catalyzed by PADI4, is an important epigenetic modification that can modulate chromatin structure and regulate gene transcription (Zhai et al., 2017). However, its function in bone homeostasis remains unclear.

We analyzed the expression profiles of *PADI4* during matrix mineralization by qPCR. The result revealed that the mRNA levels of *PADI4* dramatically increased by more than 100-fold on the third day of mineralization. Although a subsequent decline in *PADI4* mRNA expression was observed, it was relatively higher than before differentiation (Figure 4C). These finding suggest that PADI4 plays an important role in matrix mineralization. To validate the regulatory binding of miR-1301-3p to PADI4, we constructed PADI4-3′-UTR luciferase reporters containing wild-type and mutated putative binding sites of miR-1301-3p (Figure 4D). The results showed that the activity of luciferase of PADI4-3′-UTR-WT significantly decreased with miR-1301-3p mimics compared to the controls (Figure 4E). To investigate whether miR-1301-3p acts with binding sites of the 3′-UTR of PADI4, we prepared constructs of PADI4 with and without the sites. These showed that miR-1301-3p had no effect on PADI4 mRNA levels in either of the two constructs (Figure 4F). However, at the protein level, the wild-type group with the miR-1301-3p binding site was knocked down by miRNA (Figure 4G). Moreover, in hFOB 1.19 cells not transfected with PADI4, neither miR-1301-3p mimics nor inhibitor played roles on *PADI4* mRNA levels (Figure 4H). However, miR-1301-3p mimics could reduce *PADI4* protein expression levels, whereas miR-1301-3p inhibitors enhanced *PADI4* expression (Figure 4I). Finally, we systematically analyzed *PADI4* expression during osteoblast mineralization and the influence of circ8500 on this process. The results revealed that the PADI4 protein expression levels coincided with its mRNA abundance (Figures 4C,J), which was upregulated during the process. As expected, the upregulation of PADI4 could be induced by circ8500 overexpression. In addition, knocking down circ8500 resulted in the downregulation of *PADI4* (Figure 4J). In the experiments of miR-1301-3p inhibition, circ8500 knockdown or overexpression had no detectable effect on PADI4 (Figure 4K), which indicated that miR-1301-3p functions as scaffold in the regulation of PADI4 by circ8500.

These findings suggest that circ8500 could regulate the expression of *PADI4* by serving as a competing RNA for miR-1301-3p during osteoblast mineralization.

### PADI4 Upregulates RUNX2 Levels and Its Transactivation Capacity

To evaluate the function of PADI4 on osteoblast mineralization, we examined its effect on ALP activity, expression of *OCN*, and calcium deposition. The results indicated that the function of PADI4 during osteoblast mineralization is similar to circ8500 (Supplementary Figure S4). Before we explored the molecular mechanism of PADI4, we assessed its biological features. PADI4 is located in the nucleus (Nakashima et al., 2013) and regulates transcription factors, the most famous of which is p53 (Tanikawa et al., 2009). Based on this, RUNX2, the key transcription factor during osteoblast differentiation, was selected as a candidate.

**FIGURE 5:**
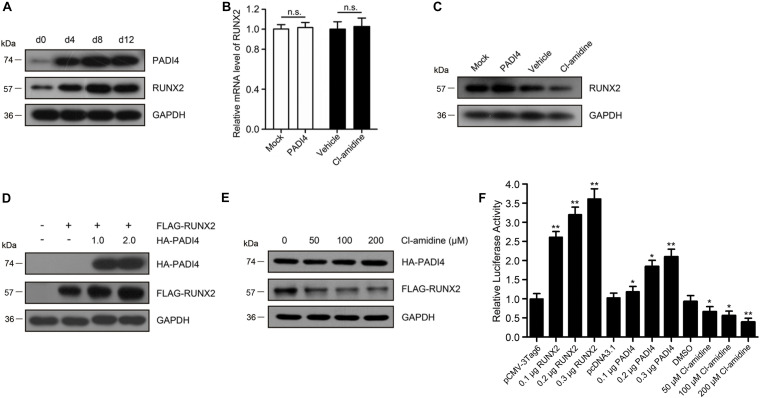
PADI4 upregulates RUNX2 levels and its transactivation capacity. **(A)** Protein expression levels of RUNX2 and PADI4 during osteoblast mineralization are examined by western blotting. **(B,C)** Relative mRNA and protein expression levels of RUNX2 were detected in hFOB 1.19 cells after transfecting with HA-PADI4, HA-NC or treated with Cl-amidine, DMSO. **(D)** HEK293T cells were transfected with FLAG-RUNX2 and increasing amounts (1.0 and 2.0 μg) of HA-PADI4. Cell lysates were immunoblotted with anti-FLAG or anti-HA antibody. **(E)** HEK293T cells were co-transfected with FLAG-RUNX2 and HA-PADI4 (0.5 μg each). 24 h later, the cells were treated with increasing amounts of Cl-amidine (50, 100, and 200 μM) for another 24 h, and the expression levels of RUNX2 and PADI4 proteins were detected. **(F)** hFOB1.19 cells were transfected with OCN-Luc reporter construct and increasing amounts of plasmids for Runx2/PADI4 or treated with increasing amounts of Cl-amidine. Cell lysates were extracted after transfection for 24 h and assayed for luciferase activity. Data are presented as the mean value ± SD (n.s., not significant, *p < 0.05, **p < 0.01).

During mineralization, PADI4 and RUNX2 share similar expression profiles (Figure 5A). We then investigated whether the regulation of RUNX2 protein expression by PADI4 occurs at the transcriptional or post-translational level. To examine this, we detected the mRNA and protein expression levels of *RUNX2* after hFOB 1.19 cells overexpressed *PADI4* or were treated with a PADI4 inhibitor. The results showed that PADI4 had no statistically indistinguishable effect on *RUNX2* transcriptional levels (Figure 5B). At the protein level, PADI4 induces the upregulation of RUNX2 (Figure 5C). These findings reveal that PADI4 regulates *RUNX2* expression at the post-translational levels. To exclude the influence of osteoblast differentiation itself and the background protein, we performed the experiments in HEK293T cells with HA-PADI4 and FLAG-RUNX2. Similarly, PADI4 induced an increase in RUNX2 protein levels, which followed a dose-dependent manner (Figure 5D). Conversely, the level of RUN2 protein expression decreased with PADI4 inhibition (Figure 5E). Cl-amidine inhibited the activity of PADI4 but did not affect protein abundance. These findings reveal that PADI4 stabilizes the RUNX2 protein, and its catalytic activity is required to mediate this effect.

We next assessed the effect of PADI4 on RUNX2 activity as a transcription factor. A luciferase reporter assay was performed in HEK293T cells using an OCN-luc reporter construct together with plasmids for RUNX2/PADI4 or with treatment of Cl-amidine. The results indicated that luciferase activity increased by RUNX2 dose-dependent. A similar up-regulatory effect was observed by ectopically expressing PADI4, although the increase was not so high. However, depletion of endogenous PADI4 protein by the inhibitor led to a reduction in luciferase activity (Figure 5F).

Together, these results suggest that PADI4 increases RUNX2 protein expression levels and its transactivation capacity.

### PADI4 Stabilizes RUNX2 via the Ubiquitin-Proteasome Pathway

**FIGURE 6:**
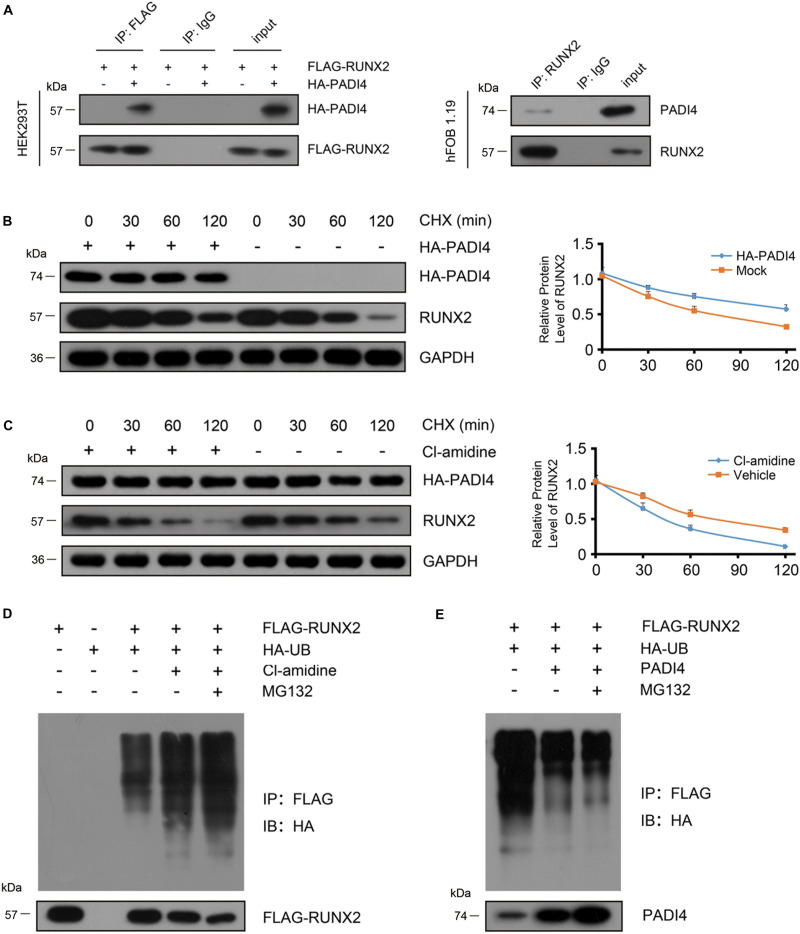
PADI4 inhibits RUNX2 degradation through the ubiquitin-proteasome pathway. **(A)** Detection of the interaction between PADI4 and RUNX2. Whole cell extracts of 293T cells co-transfected with HA-PADI4 and FLAG-RUNX2 were subjected to immunoprecipitation with anti-FLAG antibody or normal mouse IgG (negative control) and subsequently immunoblotted with antibody against HA or FLAG (left). Whole-cell extracts of hFOB1.19 cells were subjected to immunoprecipitation with anti-RUNX2 antibody or normal mouse IgG (negative control) and subsequently immunoblotted with antibody against PADI4 or RUNX2 (right). hFOB1.19 cells were stably transfected with HA-PADI4 **(B)** or treated with Cl-amidine (100 μM) for 24 h **(C)**. Then the cells were treated with cycloheximide (20 μg/mL) and subsequently harvested after 30, 60, and 120 min, respectively. Cell lysates were then immunoblotted with anti-RUNX2 or anti-PADI4 antibody. **(D)** HEK293T cells were transfected with FLAG-RUNX2 either alone or together with HA-tagged ubiquitin. Before harvest, cells were treated with Cl-amidine for 24 h and MG132 (10 μM) for 6 h. Cell lysates were immunoprecipitated with anti-FLAG antibody and subjected to immunoblotting with anti-HA antibody. **(E)** HEK293T cells were transfected with the indicted vectors (PADI4-puro-pLVX, without HA tag, was used for PADI4 transfection.) for 48 h and then treated with MG132 (10 μM) for 6 h. Cell lysates were immunoprecipitated with anti-FLAG antibody and subjected to immunoblotting with anti-HA antibody.

As shown above, cells ectopically expressing PADI4 showed a higher RUNX2 protein abundance. To further investigate how PADI4 influences RUNX2, we first overexpressed HA-PADI4 and FLAG-RUNX2 in HEK293T cells and performed co-immunoprecipitation (IP) assays to determine whether PADI4 associates with RUNX2. Figure 6A (left) shows that HA-PADI4 binds to FLAG-RUNX2. To further confirm our finding, we also investigated the interaction between endogenous PADI4 and RUNX2 in hFOB 1.19 cells by co-IP. The results using osteoblasts coincided with the physical interaction between the two proteins in HEK293T cells (Figure 6A, right).

As reported earlier, PADI4 could stabilize its binding partner (Deplus et al., 2014). To substantiate our finding that PADI4 modulates the steady state level of RUNX2, we treated hFOB1.19 cells with cycloheximide (CHX) at various time points to inhibit protein synthesis and then performed western blotting to detect endogenous RUNX2. The results showed that the degradation rate of endogenous RUNX2 significantly decrease in the presence of PADI4 (Figure 6B). When treated with Cl-amidine, which is a PADI4 inhibitor, the degradation rate of proteins was accelerated (Figure 6C). To examine whether RUNX2 degradation occurs through the ubiquitin-proteasome pathway, we performed a ubiquitination assay using osteoblast cells. We co-transfected FLAG-RUNX2 with HA-UB in hFOB 1.19 cells and then treated these with Cl-amidine or DMSO for 24 h, then RUNX2 was immunoprecipitated with anti-FLAG antibody, and polyubiquitinated RUNX2 was immunoblotted with an anti-HA antibody. The results showed that the PADI4 inhibitor promoted RUNX2 ubiquitination, and a further increase in ubiquitination of RUNX2 was observed in cells treated with MG132 (Figure 6D). When *PADI4* was overexpressed, a moderate but significant decrease in RUNX2 ubiquitination was observed (Figure 6E). These findings indicate that PADI4 stabilizes RUNX2 through the ubiquitin-proteasome pathway.

**FIGURE 7:**
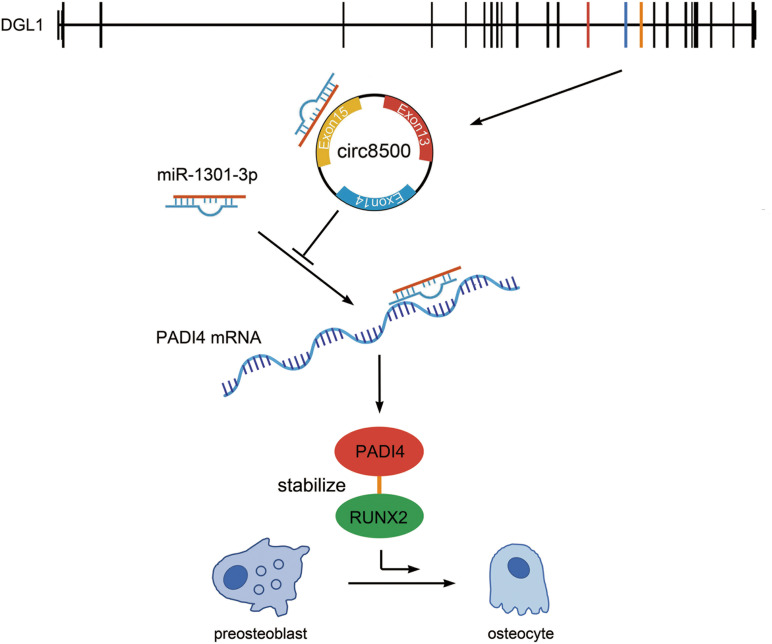
Hypothesis diagram illustrating the function and mechanism of circ8500 during osteoblast mineralization. Circ8500 is derived from exons 13, 14, and 15 of the *DGL1* gene, and cyclizes “head-to-tail” at the splicing site. Circ8500 acts as a sponge of miR-1301-3p and releases its target gene, PADI4. PADI4 interacts and stabilizes RUNX2. Finally, RUNX2 promotes osteoblast mineralization.

In summary, circ8500 exerted its function as a sponge that competitively bound to miR-1301-3p, then abolished the endogenous suppressive effect of miR-1301-3p on the target gene *PADI4*. Elevated PADI4 levels could interact with RUNX2 and stabilize it through the ubiquitin-proteasome pathway. As a key transcription factor, RUNX2 promotes osteoblast mineralization (Figure 7).

## Discussion

Studies on circRNAs can be traced back to more than 40 years ago (Hsu and Coca-Prados, 1979). Since then, circRNAs have been sporadically reported. In recent years, with advances in next-generation sequencing technologies, an increasing number of circRNAs have been identified in various tissues and cell lines. CircRNAs play regulatory roles in various biological processes and are considered as promising diagnostic markers or therapeutic targets in many diseases (Zhang et al., 2018). However, only a few circRNAs have been well characterized in bone metabolism and the biological function of circRNAs in osteoblast mineralization remains largely unknown.

In this study, we applied RNA-seq to obtain the co-expression profile of circRNAs and mRNAs in four groups of osteoblasts at different mineralization stages. Based on profile analyses of circRNAs and mRNAs, we identified circ8500 as a functional circRNA in osteoblasts. Overexpression and knockdown experiments demonstrated that circ8500 is associated with osteoblast mineralization. As a novel circRNA, there were no reports of circ8500 characterized in biological process. Our findings may shed new light on the role of circRNAs in osteoblast mineralization.

CircRNAs can work as competing endogenous RNAs (ceRNAs) to sponge miRNAs and inhibit their activity (Mitra et al., 2018). miRNAs bind to the 3′-UTR of mRNA and function in RNA silencing and post-transcriptional regulation of gene expression. In this study, we performed bioinformatics analysis to select miRNAs, of which only miR-1301-3p hit the target of circ8500. The binding of miR-301-3p to circ8500 in osteoblasts was confirmed in the subsequent experiments (Figure 3). However, miRNAs can combine with hundreds of genes in mRNAs and play different roles in different biological processes. miR-1301-3p is such a miRNA. In glioma cells, miR-1301-3p targets N-Ras and inhibits cell proliferation (Zhi et al., 2017). In prostate cancer, it promotes cell expansion by targeting SFRP1 and GSK3β (Song et al., 2018). Although it plays important roles in other disease, its function in bone metabolism has not been elucidated. In this study, we identified PADI4 as the target of miR-1301-3p because it was one of numerous predicted candidates and was detected in our mRNA sequencing data. Subsequent luciferase assays showed the binding activities of miR-1301-3p to PADI4 (Figures 4A–F). We further demonstrate that circ8500 upregulates *PADI4* by competitively binding to miR-1301-3p (Figures 4J,K).

As a peptidylarginine deiminase, PADI4 mediates citrullination, which is an important epigenetic modification. In addition, it can modulate chromatin structure and regulate gene transcription (Zhai et al., 2017). Moreover, protein citrullination also participates in transcription regulation, with the potential of antagonizing histone methylation (Cuthbert et al., 2004; Wang et al., 2004; Raijmakers et al., 2007) and coordinating histone deacetylation (Denis et al., 2009). Dysregulation of PADI4 is involved in the occurrence and development specific human diseases, such as tumor development (Chang and Fang, 2010), multiple sclerosis (Mastronardi et al., 2006), and rheumatoid arthritis (RA) (Suzuki et al., 2003; Guzmán-Guzmán et al., 2015). Several studies have revealed that PADI4 participates in cell differentiation. PADI4 is upregulated during granulocyte and monocyte differentiation in human myeloid leukemia HL-60 cells (Zhang et al., 2017). Targeting PADI4 either via RNAi or the inhibitor Cl-amidine can induce differentiation of HT29 colon cancer cells (Slack et al., 2011). In the present study, we analyzed the expression profile of PADI4 in osteoblasts (Figures 4C, 5A). More importantly, PADI4 promotes osteoblast mineralization in a way consistent with circ8500 (Figure 2 and Supplementary Figure S4). In the early stage of mineralization, ALP activity rapidly increased, whereas in the middle and late stages of mineralization, OCN secretion is accelerated. These finding suggest that circ8500 and PADI4 share similar regulatory mechanisms during mineralization. We also attemptd to elucidate the molecular mechanism of PADI4 during mineralization.

RUNX2 is the critical transcription factor in osteoblast differentiation, that can activate the transcription of several osteogenesis genes (Kern et al., 2001; Zheng et al., 2003). Considering that PADI4 could be involved in cell differentiation (Slack et al., 2011) and regulation transcriptional factors, such as p53 (Li et al., 2008), we investigated the role of PADI4 on RUNX2. First, we found that PADI4 does not influence RUNX2 mRNA abundance. Ectopically expression of PADI4 leads to an increase in *RUNX2* protein expression, whereas Cl-amidine inhibits the protein (Figures 5B,C). These results indicate that PADI4 regulates *RUNX2* protein abundance mainly at the post-translational level. Both PADI4 and RUNX2 are located in the nucleus, and thus it is possible that those two proteins interact with each other. RUNX2 was identified by co-IP assay as a novel binding partner of PADI4 (Figure 6A), and PADI4 can stabilize its binding partner (Deplus et al., 2014). We assessed the decay rate of PADI4 to RUNX2, and the results showed that PADI4 increases the stability of the RUNX2 protein (Figures 6B,C). Furthermore, *in vivo* ubiquitination assays show that PADI4 inhibits the ubiquitination of RUNX2, whereas MG132 treatment restores RUNX2 expression, indicating that PADI4 regulates ubiquitin-mediated proteasome degradation of RUNX2 (Figures 6D,E). RUNX2 is the key gene in osteoblast differentiation and its role has been well characterized.

The present study, has elucidated how circ8500-miR-1301-3p-PADI4 regulates osteoblast mineralization. However, several questions remain unclear. We confirm the interaction of PADI4 and RUNX2, but we did not investigate specific binding motifs. Cl-amidine works by inhibiting the enzyme activity of PADI4 rather than affecting its expression. This reveals that PADI4 stabilizes RUNX2 in a citrullination enzyme-dependent manner. Whether there is crosstalk between citrullination and proteasome degradation warrants further investigation.

In conclusion, our study reveals that circ8500 competitively binds to miR-1301-3p and abolishes its suppressive effect on PADI4. PADI4 stabilizes RUNX2, which promotes osteoblast mineralization. Our findings improve our understanding of the mechanism of osteoblast mineralization.

## Data Availability Statement

RNA-Seq datasets were analyzed in this study. This data can be found in Gene Expression Omnibus (GEO, Submission: GSE148203. www.ncbi.nlm.nih.gov/geo). The other original contributions presented in the study are included in the article/Supplementary Material, further inquiries can be directed to the corresponding authors.

## Author Contributions

QZ performed western blotting, co-IP, luciferase assays, and *in vivo* protein decay experiments. YZ prepared the constructs, designed the primers, and performed all of the qPCR analyses. LPW contributed to RNA isolation and osteocalcin detection. YD and PZ contributed to ALP activity detection and Von Kossa staining. XX and KL performed RIP and RNA pull down experiments. WD, WT, and BY contributed to cell culture and transfection. TL and LQW designed the project. LQW analyzed the RNA-seq data and wrote the manuscript. All authors contributed to the article and approved the submitted version.

## Conflict of Interest

The authors declare that the research was conducted in the absence of any commercial or financial relationships that could be construed as a potential conflict of interest.

## Footnotes

1 ftp://ftp.ensembl.org/pub/release-84/fasta/homo_sapiens/dna/

2 http://www.circbase.org/

3 http://regrna.mbc.nctu.edu.tw/

4 http://www.targetscan.org/vert_72
