# Supplementary material for: CircRNA hsa_circ_0008500 Acts as a miR-1301-3p Sponge to Promote Osteoblast Mineralization by Upregulating PADI4
Source: Front Cell Dev Biol. 2020 Dec 11;8:602731. doi: 10.3389/fcell.2020.602731 (PMC7759526; doi:10.3389/fcell.2020.602731)
Supplement: Supplementary file 1 [file Data_Sheet_1.PDF]

Table S1 All the Runs Used in the RNA-seq Analysis

Sample name	Raw reads	Clean reads	Clean bases	Error rate (%)	Q20 (%)	Q30 (%)	GC content (%)
**d0**	109610216	106960538	16.04G	0.01	97.77	94.19	49.67
**d3**	102097012	99626486	14.94G	0.01	97.75	94.13	49.31
**d6**	109593502	105594194	15.84G	0.01	97.38	93.31	49.26
**d9**	111320786	107180800	16.08G	0.01	97.36	93.22	50.20

Table S2 Primers used in PCR and qPCR assays

Gene Names	Primers-Forward (5' → 3')	Primers-Reverse (5' → 3')
**hsa_circ_0008500**	TGTTGCACAATATCGACCTGA	CTGCTTTAGAAACTGGGGAGT
**miR-1301-3p**	AGTATTGCAGCTGCCTGGGA	CCAGTGCAGGGTCCGAGGTA
**PADI4**	CCCAAACAGGGGGTATCAGT	CCACGGACAGCCAGTCAGAA
**DLG1**	GACCTCAGAGCTGCTAGTCAT	CTATTCATCATCTGCTCCCGT
**RUNX2**	GTAGCAAGGTTCAACGATCTGA	TCCCGAGGTCCATCTACTGTAA
**U6**	CGCTTCGGCAGCACATATAC	CAGGGGCCATGCTAATCTT
**GAPDH (qPCR)**	AGCCACATCGCTCAGACACCA	ATGTAGTTGAGGTCAATGAA
**Divergent**	GACCTCAGAGCTGCTAGTCA	TCATCATCTCCAAGTACTGCT
**Convergent**	AGACACCAGCATCTCCAGCCA	GCTCATGACTAGCAGCTCTGA
**GAPDH (PCR)**	TGTTCCAATATGATTCCACCC	GGTTCACACCCATGACGAACA

Table S3 Primers used in the recombined plasmids

Constructs	Templates	Primer-Forward (5'→3')	Primer-Forward (5'→3')	Cloning sites	Cloning Elements & Target Vectors
pLVX-CircRNA	pcDNA3.1(+) CircRNA Mini	AGTCGAATTCAAAGTGCTGAGATTACAGGC	AGTCGGATCCTGCTGGGATTACAGGTGTGAG	*Eco*R I, *Bam*H I	AluSq2 and AluSz; pLVX-puro
pcDNA3.1-Circ8500	hFOB1.19 cDNA	CTTCTTCTCAGCCTGTTGATAACC	AGTACCGCGGCTTCAGGTCGATATTGTGCA	*Eco*R V, *Sac* II	hsa_circ_0008500; pcDNA-3.1(+) CircRNA Mini
pLVX-Circ8500	pcDNA3.1-Circ8500	AGTCGAATTCAAAGTGCTGAGATTACAGGC	AGTCGGATCCTGCTGGGATTACAGGTGTGAG	*Eco*R I, *Bam*H I	AluSq2 and AluSz, hsa_-circ_0008500; pLVX-puro
pLKO.1-Circ8500	-	CCGGTCGACCTGAAGCTTCTTCTCAGCTCGAGCTGAGAAGAAGCTTCAGGTCGATTTTTG	AATTCAAAAATCGACCTGAAGCTTCTTCTCAGCTCGAGCTGAGAAGAAGCTTCAGGTCGA	*Eco*R I, *Age* I	hsa_circ_0008500 inter-ference sequence; pLKO.1
pLKO.1-NC	-	CCGGTTCTCCGAACGTGTCACGTTTCCTCGAG GAAACGTGACACGTTCGGAGAATTTTTG	AATTCAAAAATTCTCCGAACGTGTCACGTTTCCTCGAG GAAACGTGACACGTTCGGAGAA	*Eco*R I, *Age* I	scrambled sequence; pLKO.1
pGL3-cir8500-WT	hFOB1.19 cDNA	GCCGTGTAATTCTAGCTTCTTCTCAGCCTG	CCGCCCCGACTCTAGCTTCAGGTCGATATT	*Xba* I	hsa_circ_0008500 transcript; pGL3-Promotor
pGL3-cir8500-Mut	Circ8500 mu-tant transcript	GCCGTGTAATTCTAGCTTCTTCTCAGCCTG	CCGCCCCGACTCTAGCTTCAGGTCGATATT	*Xba* I	hsa_circ_0008500 mutant transcript; pGL3-Promotor
pGL3-PADI4-WT	hFOB1.19 cDNA	GCCGTGTAATTCTAGGCCCATCTTCCCTGG	CCGCCCCGACTCTAGTCATGTACAAAGTGA	*Xba* I	PADI4 3'-UTR; pGL3-Promotor
pGL3-PADI4-Mut	PADI4 mutant	GCCGTGTAATTCTAGGCCCATCTTCCCTGG	CCGCCCCGACTCTAGTCATGTACAAAGTGA	*Xba* I	PADI4 mutant 3'-UTR; pGL3-Promotor
pcDNA3.1-PADI4-3'-UTR WT	hFOB1.19 cDNA	AGTCGGATCCATGGCCCAGGGGACATTGAT	AGTCGAATTCTCATGTACAAAGTGACTTA	*Bam*H I, *Eco*R I	PADI4 ORF with WT 3'-UTR; pcDNA3.1 (+)

PADI4-3'-UTR mut	PADI4-3'-UTR WT	GTTCTGCTCTAAGAAATAAAGTTTTTTTAA	TTAAAAAAACTTTATTTCTTAGAGCAGAAC	-	PADI4 ORF with mut 3'-UTR; pcDNA3.1 (+)
PADI4-pLVX-puro	hFOB1.19 cDNA	GACTGAATTCGCCACCATGGCCCAGGGGACATTGAT	GACTGGATCCTCAGGGCACCATGTTCCACCAC	*Eco*R I, *Bam*H I	PADI4 ORF; pLVX-puro
pCMV-3×FLAG-RUNX2	hFOB1.19 cDNA	TGACAGATCTATGGCATCAAACAGCCTCT	TGACCTCGAGTCAATATGGTCGCCAAACAG	*Bam*H I /*Bgl* II, *Xho* I	RUNX2 ORF; pCMV-3Tag6

Table S4 The predicted top 50 target genes for miR-1301-3p

No.	Target gene	Site counts				6mer sites	Cumulative weighted context ++ score	Total context ++ score
		total	8mer	7mer -m8	7mer- A1			
1	TFRC	**4**	2	1	1	1	-1.33	-1.34
2	RP11-738G5.2	**1**	1	0	0	0	-0.96	-0.96
3	TRIAP1	**2**	1	0	1	1	-0.91	-0.93
4	NDUFB10	**2**	1	0	1	0	-0.91	-0.91
5	RP5-850E9.3	**2**	2	0	0	0	-0.87	-0.87
6	SRXN1	**2**	2	0	0	0	-0.84	-0.84
7	RUFY1	**5**	1	4	0	0	-0.82	-0.82
8	RP11-664D7.4	**2**	1	0	1	4	-0.82	-0.91
9	C1orf65	**1**	1	0	0	0	-0.81	-0.81
10	CDCP2	**2**	2	0	0	1	-0.76	-0.76
11	APOD	**1**	1	0	0	1	-0.75	-0.75
12	TBC1D21	**1**	1	0	0	0	-0.75	-0.75
13	PIF1	**2**	1	1	0	1	-0.74	-0.74
14	SSBP3-AS1	**2**	1	0	1	1	-0.71	-0.71
15	RHOA	**2**	1	0	1	0	-0.70	-0.70
16	SASH3	**2**	2	0	0	0	-0.69	-0.69
17	TCP11X2	**1**	1	0	0	0	-0.69	-0.69
18	TCP11X1	**1**	1	0	0	0	-0.69	-0.69
19	AC119673.1	**1**	1	0	0	0	-0.68	-0.68
20	RP11-215A19.2	**1**	1	0	0	0	-0.68	-0.68
21	RNF38	**4**	1	1	2	0	-0.65	-0.70
22	C10orf99	**2**	1	0	1	0	-0.65	-0.65
23	ANAPC11	**1**	1	0	0	0	-0.64	-0.64
24	PADI4	**1**	1	0	0	0	-0.64	-0.64
25	G3BP2	**2**	1	0	1	1	-0.64	-0.64

26	DNAJC27	**3**	2	1	0	1	-0.64	-0.76
27	ARL5A	**2**	1	1	0	0	-0.63	-0.77
28	C17orf58	**1**	1	0	0	0	-0.63	-0.63
29	HAUS5	**5**	0	3	2	1	-0.62	-0.62
30	FDXR	**1**	1	0	0	1	-0.61	-0.61
31	KMT2D	**2**	1	0	1	1	-0.61	-0.62
32	MT1M	**1**	0	1	0	1	-0.60	-0.60
33	FDX1L	**1**	1	0	0	2	-0.59	-0.70
34	MARCKSL1	**1**	1	0	0	0	-0.59	-0.59
35	HS6ST3	**2**	2	0	0	4	-0.59	-0.60
36	RHEB	**1**	1	0	0	0	-0.59	-0.59
37	LUZP6	**1**	1	0	0	0	-0.59	-0.59
38	PAQR5	**2**	1	1	0	1	-0.59	-0.69
39	LMNB1	**1**	1	0	0	0	-0.59	-0.60
40	SKIL	**2**	1	1	0	0	-0.59	-0.64
41	DGCR2	**4**	0	3	1	5	-0.58	-0.59
42	BPIFB2	**1**	1	0	0	0	-0.58	-0.58
43	TMEM150A	**2**	0	2	0	1	-0.58	-0.60
44	IGSF5	**1**	1	0	0	0	-0.58	-0.58
45	EVA1B	**2**	0	1	1	0	-0.58	-0.58
46	ADAM21	**1**	1	0	0	0	-0.57	-0.57
47	HOXC4	**1**	1	0	0	0	-0.57	-0.57
48	C14orf23	**1**	1	0	0	2	-0.57	-0.62
49	RELL1	**1**	1	0	0	2	-0.56	-0.56
50	HEBP1	**1**	1	0	0	0	-0.56	-0.56
