# Supplementary material for: CircRNA hsa_circ_0008500 Acts as a miR-1301-3p Sponge to Promote Osteoblast Mineralization by Upregulating PADI4
Source: Front Cell Dev Biol. 2020 Dec 11;8:602731. doi: 10.3389/fcell.2020.602731 (PMC7759526; doi:10.3389/fcell.2020.602731)
Supplement: Supplementary file 2 [file Presentation_1.PPTX]

## Slide 1
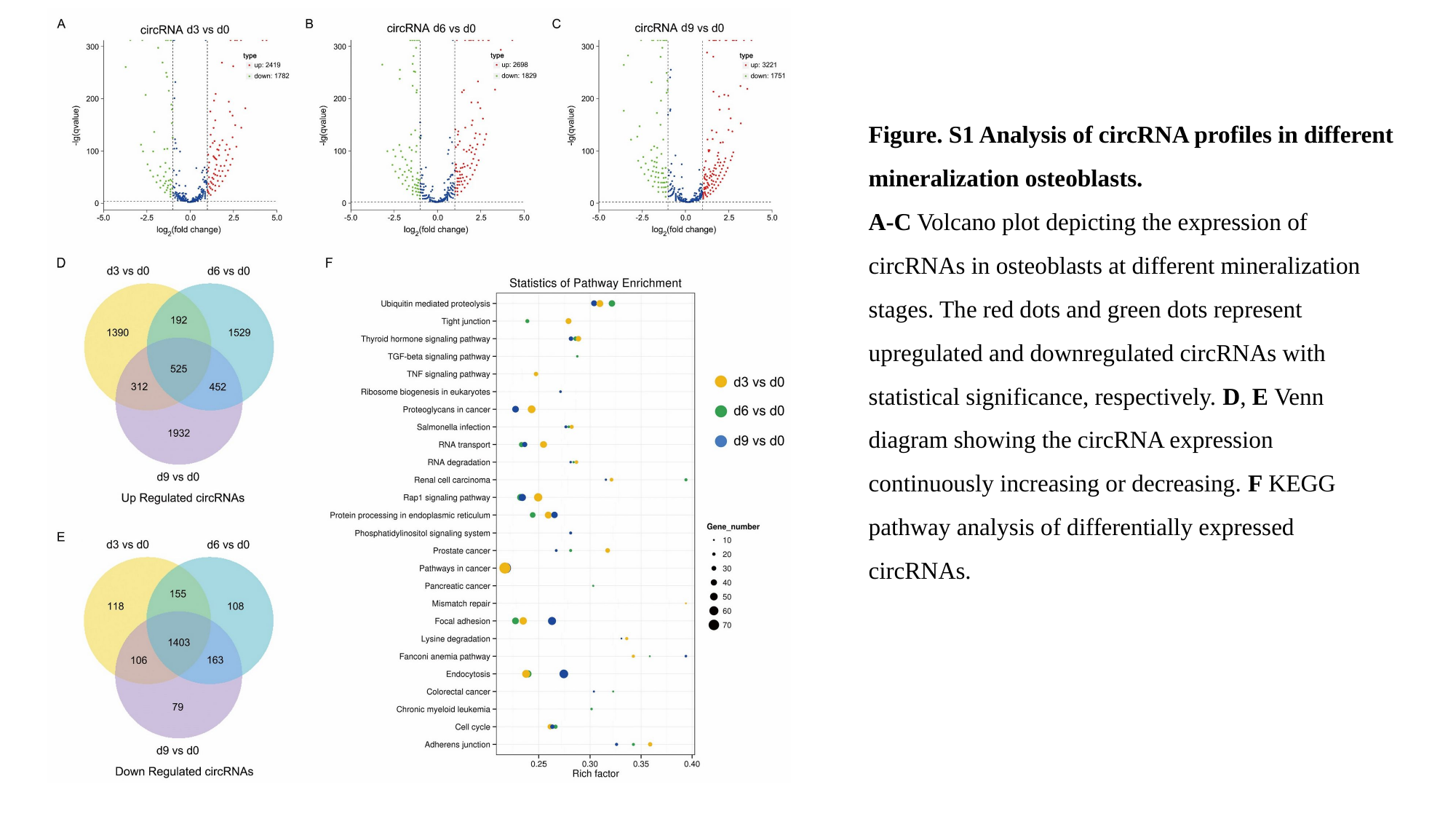

Figure. S1 Analysis of circRNA profiles in different mineralization osteoblasts.
A-C Volcano plot depicting the expression of circRNAs in osteoblasts at different mineralization stages. The red dots and green dots represent upregulated and downregulated circRNAs with statistical significance, respectively. D, E Venn diagram showing the circRNA expression continuously increasing or decreasing. F KEGG pathway analysis of differentially expressed circRNAs.

## Slide 2
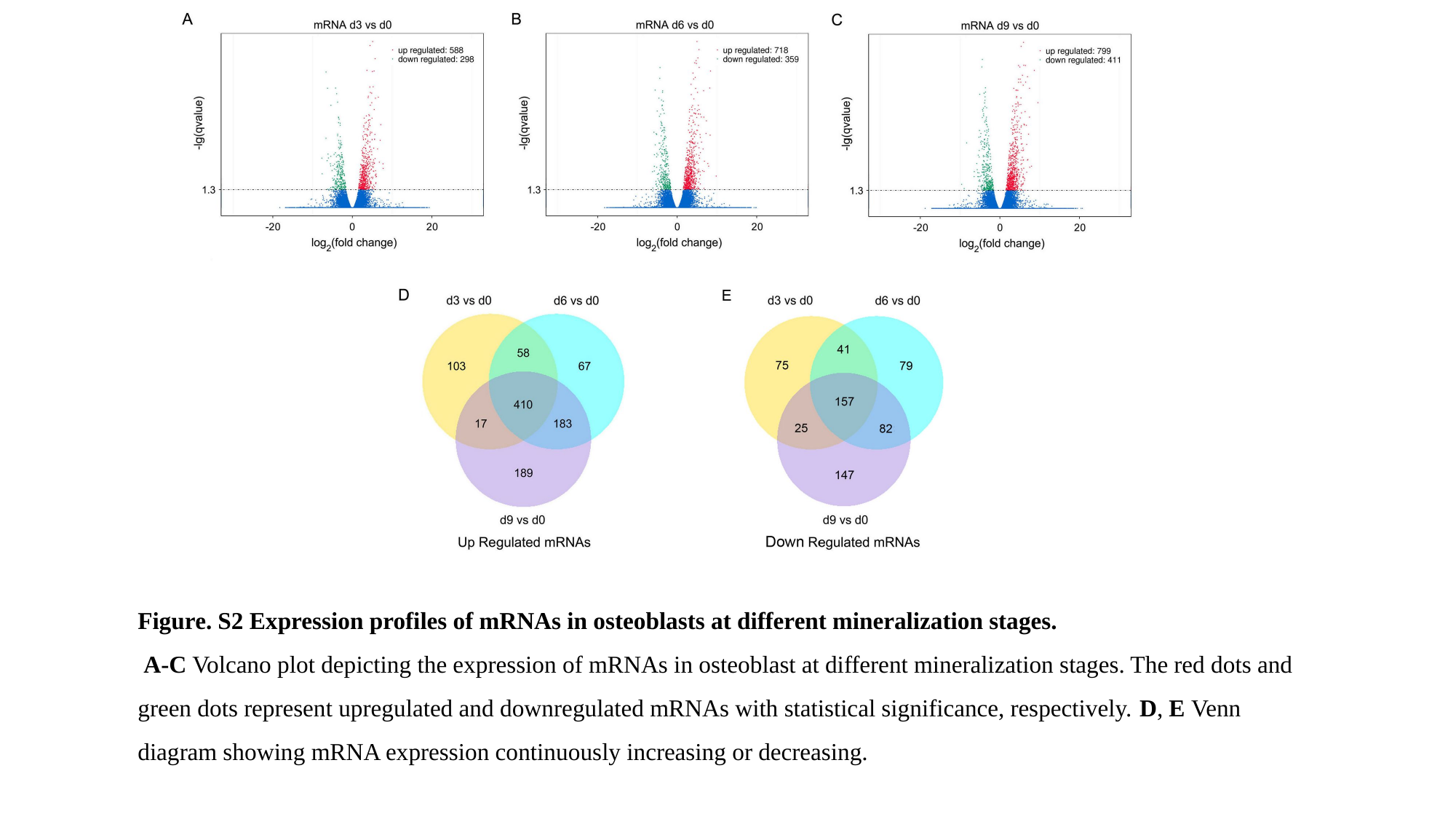

Figure. S2 Expression profiles of mRNAs in osteoblasts at different mineralization stages.
 A-C Volcano plot depicting the expression of mRNAs in osteoblast at different mineralization stages. The red dots and green dots represent upregulated and downregulated mRNAs with statistical significance, respectively. D, E Venn diagram showing mRNA expression continuously increasing or decreasing.

## Slide 3
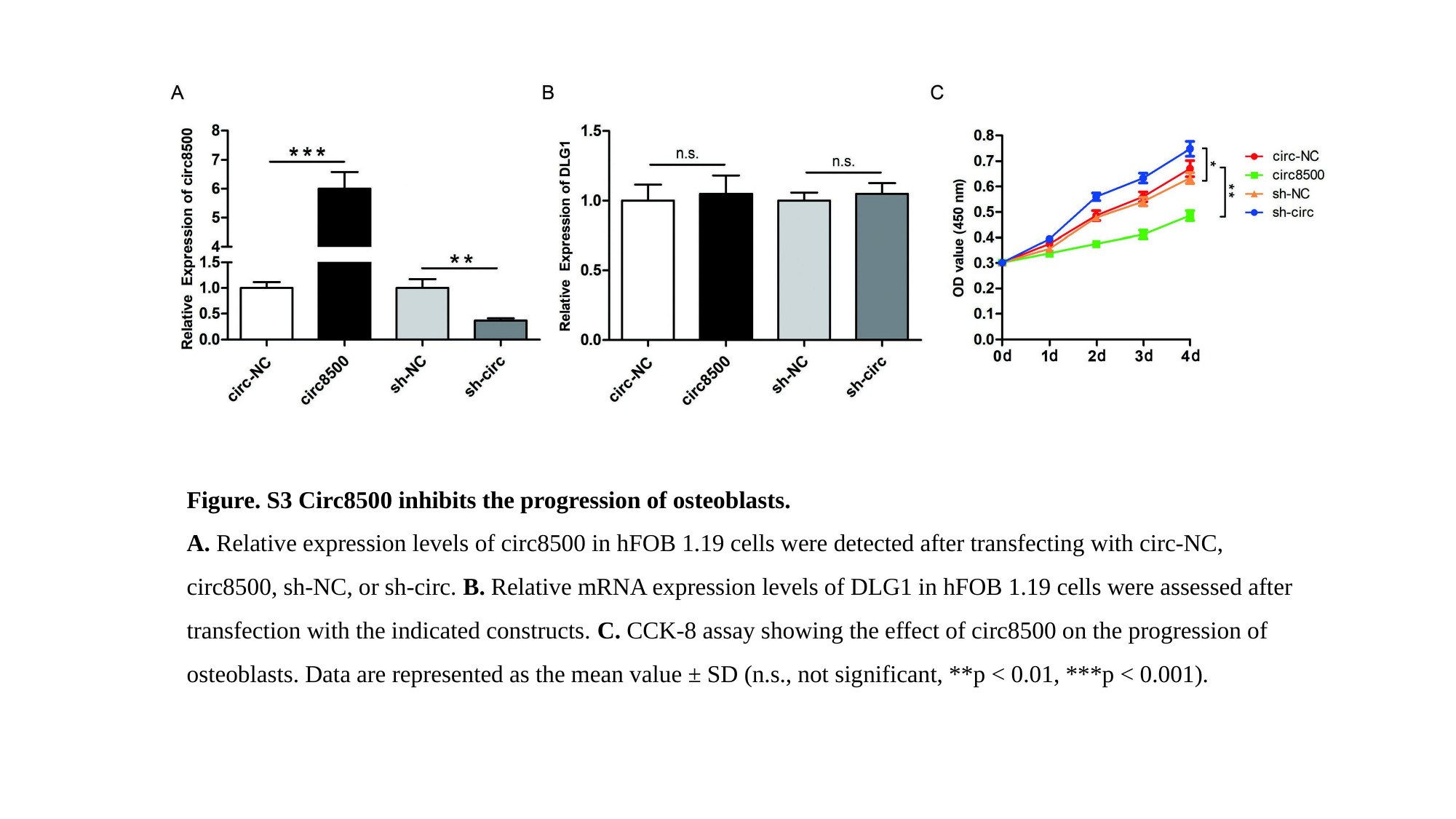

Figure. S3 Circ8500 inhibits the progression of osteoblasts.
A. Relative expression levels of circ8500 in hFOB 1.19 cells were detected after transfecting with circ-NC, circ8500, sh-NC, or sh-circ. B. Relative mRNA expression levels of DLG1 in hFOB 1.19 cells were assessed after transfection with the indicated constructs. C. CCK-8 assay showing the effect of circ8500 on the progression of osteoblasts. Data are represented as the mean value ± SD (n.s., not significant, **p < 0.01, ***p < 0.001).

## Slide 4
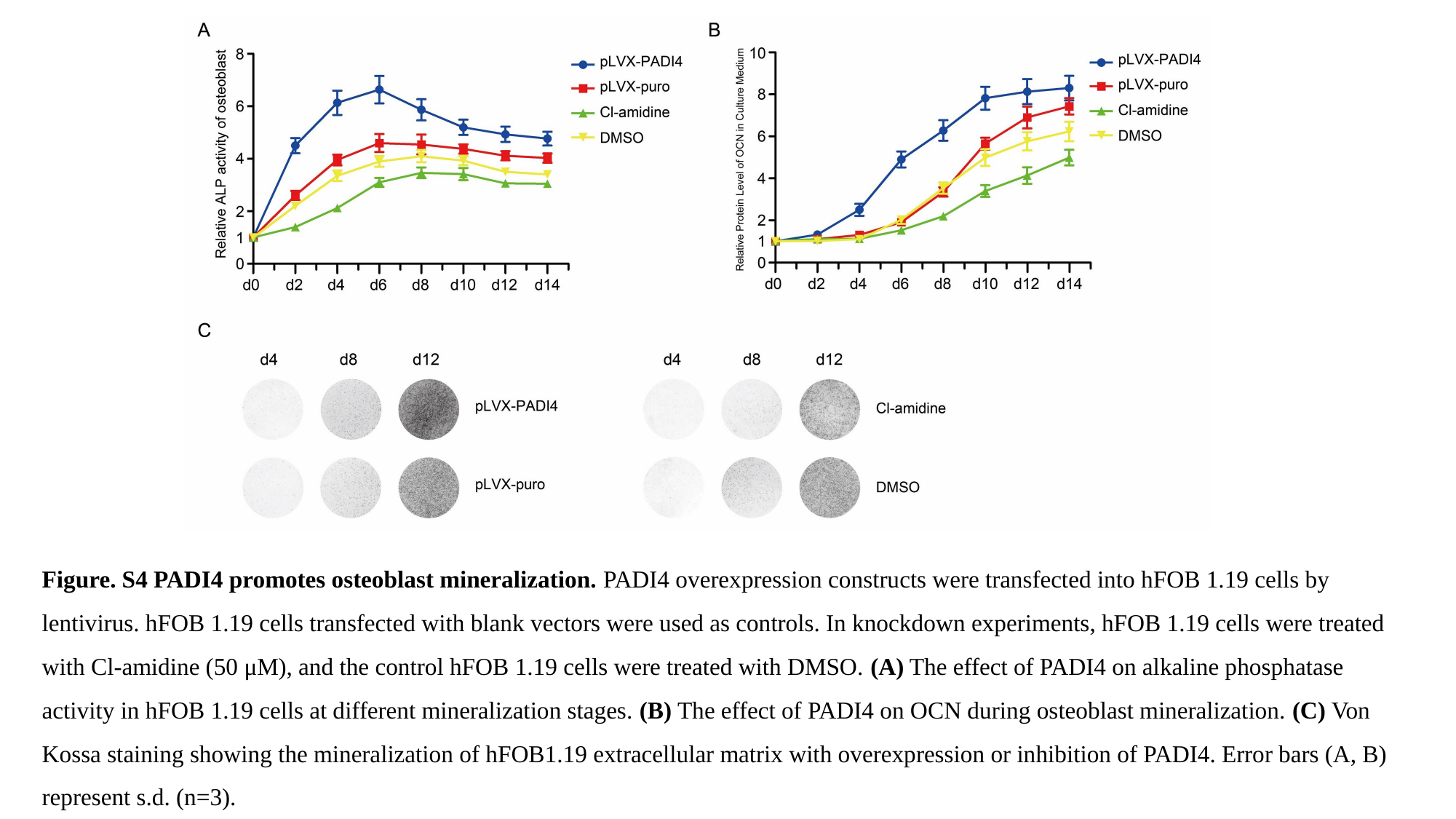

Figure. S4 PADI4 promotes osteoblast mineralization. PADI4 overexpression constructs were transfected into hFOB 1.19 cells by lentivirus. hFOB 1.19 cells transfected with blank vectors were used as controls. In knockdown experiments, hFOB 1.19 cells were treated with Cl-amidine (50 μM), and the control hFOB 1.19 cells were treated with DMSO. (A) The effect of PADI4 on alkaline phosphatase activity in hFOB 1.19 cells at different mineralization stages. (B) The effect of PADI4 on OCN during osteoblast mineralization. (C) Von Kossa staining showing the mineralization of hFOB1.19 extracellular matrix with overexpression or inhibition of PADI4. Error bars (A, B) represent s.d. (n=3).
